# Diagnostic performance of measuring antibodies to the glycopeptidolipid core antigen specific to *Mycobacterium avium* complex in patients with rheumatoid arthritis: results from a cross-sectional observational study

**DOI:** 10.1186/s13075-015-0787-y

**Published:** 2015-09-28

**Authors:** Wataru Hirose, Takashi Uchiyama, Asuka Nemoto, Masayoshi Harigai, Kenji Itoh, Toshiaki Ishizuka, Mitsuyo Matsumoto, Kazue Yamaoka, Toshihiro Nanki

**Affiliations:** Hirose Clinic of Rheumatology, 2-14-7, Midori-cho, Tokorozawa city, Saitama 359-1111 Japan; Division of Respiratory Medicine, Fukujuji Hospital, Japan Anti-Tuberculosis Society, 3-1-24, Matsuyama, Kiyose city, Tokyo 204-8522 Japan; Graduate School of Public Health, Teikyo University, 2-11-1, Kaga, Itabashi-ku, Tokyo 173-8605 Japan; Department of Pharmacovigilance, Graduate School of Medical and Dental Sciences, Tokyo Medical and Dental University, 1-5-45, Yushima, Bunkyo-ku, Tokyo 113-8519 Japan; Division of Rheumatology, National Defense Medical College, 3-2, Namiki, Tokorozawa city, Saitama 359-0042 Japan; Department of Pharmacology, National Defense Medical College, 3-2, Namiki, Tokorozawa city, Saitama 359-0042 Japan; Department of Clinical Research Medicine, Teikyo University, 2-11-1, Kaga, Itabashi-ku, Tokyo 173-8605 Japan; Division of Rheumatology, Department of Internal Medicine, Toho University School of Medicine, 6-11-1, Omori-Nishi, Ota-ku, Tokyo 143-8541 Japan

## Abstract

**Introduction:**

The aim of this study was to investigate the diagnostic performance of measuring antibodies to the glycopeptidolipid (GPL) core antigen specific to *Mycobacterium avium* complex (MAC) in patients with rheumatoid arthritis (RA).

**Methods:**

We cross-sectionally investigated anti-GPL antibodies and radiographs of 396 patients with RA. A diagnosis of MAC pulmonary disease (MAC-PD) was made according to the criteria by the American Thoracic Society and the Infectious Diseases Society of America. Serum immunoglobulin A antibodies to MAC-specific GPL core antigen were measured by an enzyme immunoassay. All patients with RA with abnormal shadows on chest x-rays underwent chest computed tomography (CT). Bronchoscopy was performed on patients with negative cultures for MAC by expectorated sputum and positive CT findings compatible with MAC-PD.

**Results:**

Ten patients were newly diagnosed with MAC-PD. Eight individuals who already had diagnoses of MAC-PD at the time of enrollment and nineteen who had negative expectorated sputum cultures for MAC and positive CT images compatible with MAC-PD and who refused bronchoscopy were excluded from the following analysis. Anti-GPL antibodies were detected in 12 of 369 patients. Eight of the ten patients with MAC-PD and 4 of 359 patients without MAC-PD tested positive for the anti-GPL antibodies. The specificity and sensitivity were 99 % and 80 %, respectively. Positive and negative predictive values were 67 %, and 97 %, respectively. When we analyzed diagnostic performance of the antibodies in 57 patients with RA who had abnormal shadows on chest x-rays, the positive and negative predictive values were 100 %, and 96 %, respectively. Twelve patients underwent bronchoscopy. Bronchoalveolar lavage fluid (BALF) samples from six patients were positive for MAC, and BALF samples from the remainder were negative. Anti-GPL antibodies were detected in the sera of all six patients with positive results for MAC by BALF sampling, whereas the antibodies were not detected in the sera from the remainder with negative results for MAC by BALF sampling.

**Conclusions:**

The measurement of anti-GPL antibodies is useful as a supplementary diagnostic tool for MAC-PD in patients with RA and may provide a new strategy, in combination with chest x-ray and CT, for differentiating MAC-PD from other pulmonary comorbidities in patients with RA.

**Electronic supplementary material:**

The online version of this article (doi:10.1186/s13075-015-0787-y) contains supplementary material, which is available to authorized users.

## Introduction

Although the emergence of biologic disease-modifying antirheumatic drugs (DMARDs) has markedly changed the course of rheumatoid arthritis (RA) and outcomes for patients, concerns have been raised regarding the higher risk of infection. Researchers in recent studies have reported an increase in the prevalence of diseases caused by nontuberculous mycobacteria (NTM) [1–4]. Eighty percent of patients with NTM diseases in Japan have been infected with *Mycobacterium avium* complex (MAC) [5]. MAC is now widely recognized as an important pathogen that causes chronic and progressive pulmonary diseases in both immunocompetent and immunosuppressed patients.

The diagnosis of MAC-PD is complicated because, in contrast to *Mycobacterium tuberculosis*, the contamination of clinical specimens by MAC can come from environmental sources such as water, dust, and soil, and this organism may colonize the respiratory tract without any accompanying invasive disease [6]. A diagnosis of MAC-PD requires clinical findings and its repeated isolation from sputum. Difficulties have also been associated with discriminating MAC-PD from rheumatoid lung disease in a clinical setting, because RA could develop pulmonary manifestations, including rheumatoid nodules, cryptogenic organizing pneumonia, bronchiolitis obliterans, and bronchiectasis [7, 8]. The most frequent finding by computed tomography (CT) in patients with RA in one study was bronchiectasis [8]. Although only 1–3 % of patients with RA clinically exhibit bronchiectasis, as many as 30 % of patients with RA manifest bronchiectasis on high-resolution computed tomography (HRCT) [9]. Bronchiectasis is considered to be both a risk factor for and a consequence of NTM infections [9]. Kitada et al*.* [6, 10] recently established an enzyme immunoassay (EIA) for the serological diagnosis of MAC-PD by examining serum immunoglobulin A (IgA) antibody levels against the glycopeptidolipid (GPL) core antigen, which is a MAC-specific antigen. Unlike bronchoscopy and sputum culture examinations, this test is less invasive and provides more rapid diagnostic information on MAC-PD.

In the present study, we focused on MAC-PD in patients with RA and conducted a cross-sectional observational study to investigate the clinical usefulness of measuring anti-GPL antibodies in this patient population.

## Methods

### Patients

A cross-sectional observational study was conducted. The study sample consisted of 396 patients who were treated for RA between May and October 2013 at the Hirose Clinic of Rheumatology, which is an outpatient clinic located in Saitama prefecture in Japan. The inclusion criteria for patients were fulfillment of the 2010 American College of Rheumatology/European League against Rheumatism classification criteria for RA [11]. Patients younger than 20 years of age and those who had already been enrolled in a clinical study with the intervention of a study drug were excluded. A total of 824 patients with RA visited our clinic during the study period. We randomly screened a total of 443 of 824 patients with RA, and a total of 381 patients with RA (46.2 %) were not screened.

Sera from all participants were screened for anti-GPL IgA antibodies, and all the participants underwent chest radiography. For statistical analyses, recorded data, including demographics, disease activity, comorbidities, treatments, and laboratory data, were obtained at the time of consent. All participants provided written informed consent before study enrollment according to the Declaration of Helsinki (World Medical Association General Assembly, October 2008). This study was conducted with the approval of the Hattori Clinic Institutional Review Board (Hachioji, Tokyo, Japan), which was responsible for reviewing and approving the study.

### Diagnosis of MAC-PD

We defined MAC-PD according to the 2007 diagnostic criteria for NTM lung disease proposed by the American Thoracic Society and Infectious Diseases Society of America [1]. Both of the following criteria had to be met clinically: (1) pulmonary symptoms, nodular or cavitary opacities on chest radiographs, or HRCT scans manifesting multifocal bronchiectasis with multiple small nodules; and (2) appropriate exclusion of other diagnoses. Only one of the following criteria was required microbiologically: (1) positive culture results for at least two separate expectorated sputum samples, (2) positive culture results for at least one bronchial wash or lavage, or (3) transbronchial or other lung biopsy specimens with mycobacterial histopathological features. A diagnosis of MAC-PD infection required the fulfillment of the clinical and microbiological criteria described above.

### Enzyme immunoassay for anti-GPL antibodies

All serum samples were sent to the Life Science Institute, Inc. (Tokyo, Japan), where a commercially available EIA kit used to detect serum IgA antibodies specific to the MAC-GPL core antigen (Capilia MAC Antibody ELISA; TAUNS Laboratories, Shizuoka, Japan) was employed with a previously described method [6]. The GPL core antigen is a major cell surface antigen in MAC that is absent on the cell wall of either *M. tuberculosis* or *Mycobacterium kansasii* [10]. At the Life Science Institute, the cutoff value was defined as 0.7 U/ml according to previous findings [6].

### Radiological evaluation

Chest x-rays were assessed by consensus reading by four rheumatologists and one respiratory physician (WH, KI, MM, TN, TU). All patients with RA who had abnormal shadows on their chest x-rays underwent chest CT. Chest CT images from patients with RA were reviewed with consensus reading by one rheumatologist and one respiratory physician experienced in CT (WH, TU). To localize the infection by MAC, the lungs of each patient were divided into 10 fields (right lung: S1 + S2, S3, S4 + S5, S6, and S7 + S8 + S9 + S10; left lung: S1 + S2, S3, S4 + S5, S6, and S8 + S9 + S10) according to Moore’s definition [12]. Each field was scored with reference to the presence of bronchiectasis, centrilobular nodules, air space disease, a cavity, and nodules larger than 5 mm as described in a previous study [13]. The severity of bronchiectasis was categorized as grade 1 (diameter of the bronchus less than twice as large as the accompanying vessel), grade 2 (diameter of the bronchus at least twice as large as the accompanying vessel), or grade 3 (cystic bronchiectasis). The distribution of centrilobular nodules (size less than 5 mm) was categorized as grade 1 (0–50 % of the segment), grade 2 (50–100 % of the segment), or grade 3 (100 % of the segment). Air space disease was defined as an area of patchy or dense consolidation. Air space disease and cavity formation were categorized as grade 3 on the basis of a previous study in which these two findings were closely related to clinical features [14]. Nodular sizes larger than 5 mm were also scored as grade 1. Grades of the 10 fields were summed to calculate scores for these radiological findings. Thus, the maximum scores of bronchiectasis, centrilobular nodules, air space disease, cavities, and nodules larger than 5 mm in both lungs were 30, 30, 30, 30, and 10, respectively. Scores for the extent of MAC-PD were added to give total CT scores [13]. Thus, the maximum possible total CT score in both lungs was 130.

### Sputum

All patients with RA with abnormal findings on chest CT compatible with MAC-PD submitted expectorated sputum for examination on three consecutive days. An early-morning specimen was collected in a sterile cup, and it was immediately transported to the microbiology laboratory at the Life Science Institute. The sputum was examined and cultured for mycobacteria. Identification of MAC was confirmed by TaqMan polymerase chain reaction (Applied Biosystems, Foster City, CA, USA) at the same laboratory. The patients whose expectorated sputum was negative for MAC were recommended to undergo bronchoscopy at the Fukujuji Hospital, which was performed once the patient’s consent was obtained.

### Statistical analysis

All data were analyzed using SAS software version 9.3 (SAS Institute, Cary, NC, USA). Demographics and clinical characteristics were expressed as means and standard deviations for continuous variables or as frequencies and percentages for categorical variables. Comparisons between patients with RA with and without MAC-PD were made using Student’s *t* test for continuous variables. Pearson’s χ^2^ test was used for categorized variables. All *P* values were two-tailed, and *P* values less than 0.05 were considered significant. The Mann–Whitney *U* test was used to compare the differences in titers of anti-GPL antibodies between patients with and those without MAC organisms in bronchoalveolar lavage fluid (BALF) samples. Correlation coefficients between the extent of MAC-PD on chest CT images and titers of anti-GPL IgA antibodies were analyzed using a simple regression analysis.

## Results

### Diagnosis of MAC-PD in enrolled patients

**Fig. 1:**
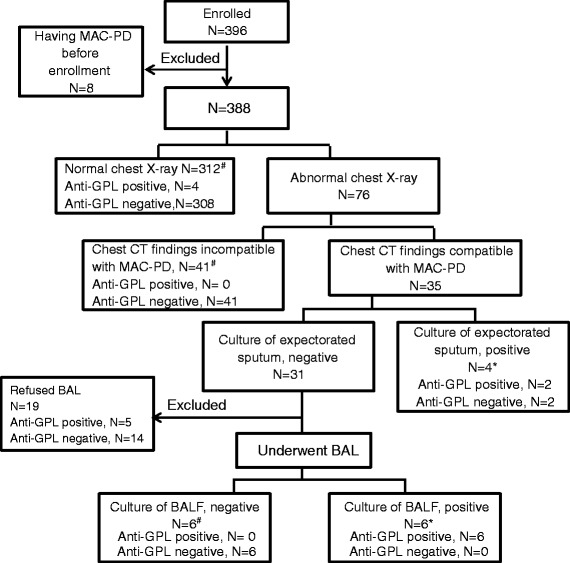
Patient disposition flow chart. *Patients in this study who were diagnosed with *Mycobacterium avium* complex pulmonary disease (MAC-PD) (n = 10 in total). #Patients in this study who were not diagnosed as having MAC-PD (n = 359 in total). *BAL* bronchoalveolar lavage, *BALF* bronchoalveolar lavage fluid, *GPL* glycopeptidolipid

Because the level of anti-GPL antibodies has been reported to reflect disease activity of MAC infection and to decrease after successful antimicrobial chemotherapy [10], eight patients who already had diagnoses of MAC-PD at the time of enrollment were excluded from the following analyses. A total of 388 patients were screened using chest x-rays, and 76 of these patients were found to have abnormal shadows. These patients underwent chest CT. Thirty-five of the seventy-six patients had abnormal CT images compatible with MAC-PD, and the remaining forty-one patients had abnormal CT images incompatible with MAC-PD. The 35 patients with abnormal CT images compatible with MAC-PD submitted expectorated sputum. Four of these patients had positive cultures for MAC and were diagnosed as having MAC-PD; the others’ cultures were negative. Bronchoscopy could be performed in 12 of the 31 patients who had negative expectorated sputum cultures for MAC and positive CT findings. The BALF of 6 of these 12 patients were positive for MAC, and these 6 patients were diagnosed as having MAC-PD; the others’ BALF samples were negative for MAC. The remaining 19 of the 31 patients who had negative expectorated sputum cultures for MAC and positive CT images compatible with MAC-PD refused bronchoscopy. Five of these nineteen patients tested positive for anti-GPL antibodies, and the remaining fourteen tested negative for these antibodies. We could not determine the presence or absence of MAC-PD in these patients and excluded them from the following statistical analyses. Consequently, we analyzed 369 patients, including 10 with and 359 without MAC-PD. Figure 1 describes the patient disposition from screening until diagnosis of MAC-PD.

### Diagnostic performance of anti-GPL antibodies

Of the 369 patients, 12 tested positive for the anti-GPL antibodies. Of the 12 anti-GPL antibody–positive patients, 8 were diagnosed with MAC-PD. Of the 357 anti-GPL antibody–negative patients, 2 were diagnosed with MAC-PD. The sensitivity, specificity, and accuracy of the assay were 80 %, 99 %, and 98 %, respectively (Table 1). On the basis of these data, the positive and negative likelihood ratios of the test were estimated as 80.0 and 0.20, respectively. A pretest probability of MAC-PD in this sample was 2.7 %. Positive and negative posttest probabilities were calculated at 69 % and 0.54 %, respectively. Positive predictive value was 67 %, and negative predictive value was 97 %. Receiver operating characteristic curves constructed for patients with and without MAC-PD revealed an area under the curve (AUC) of 0.962.

**Table 1:** Results of screening of sera for anti-GPL antibodies from 369 patients with rheumatoid arthritis

	RA with MAC-PD	RA without MAC-PD	Total
Anti-GPL ab, positive	8	4	12
Anti-GPL ab, negative	2	355	357
Total	10	359	369

*Abbreviations: ab* antibodies, *GPL* glycopeptidolipid, *MAC Mycobacterium avium* complex *PD* pulmonary disease, *RA* rheumatoid arthritis

Variables are the number of patients unless otherwise indicated

We also analyzed the diagnostic performance of anti-GPL antibodies in patients with RA who had abnormal chest x-rays. We excluded 19 patients who had positive CT images compatible with MAC-PD and negative expectorated sputum cultures for MAC and who refused bronchoscopy; thus, 57 of 76 patients with abnormal chest x-ray findings were subjected to this analysis. The sensitivity, specificity, and accuracy of the assay in these patients were 80 %, 100 %, and 97 %, respectively (Table 2). Positive and negative predictive values were calculated at 100 % and 96 %, respectively.

**Table 2:** Diagnostic performance of anti-GPL antibodies in 57 patients with rheumatoid arthritis with abnormal shadows on chest x-rays

	RA with MAC-PD	RA without MAC-PD	Total
Anti-GPL ab, positive	8	0	8
Anti-GPL ab, negative	2	47	49
Total	10	47	57

*Abbreviations: ab* antibodies, *GPL* glycopeptidolipid, *MAC Mycobacterium avium* complex *PD* pulmonary disease, *RA* rheumatoid arthritis

Variables are the number of patients unless otherwise indicated

### Relationship between chest CT findings and titers of anti-GPL antibodies in patients with RA and MAC-PD

**Fig. 2:**
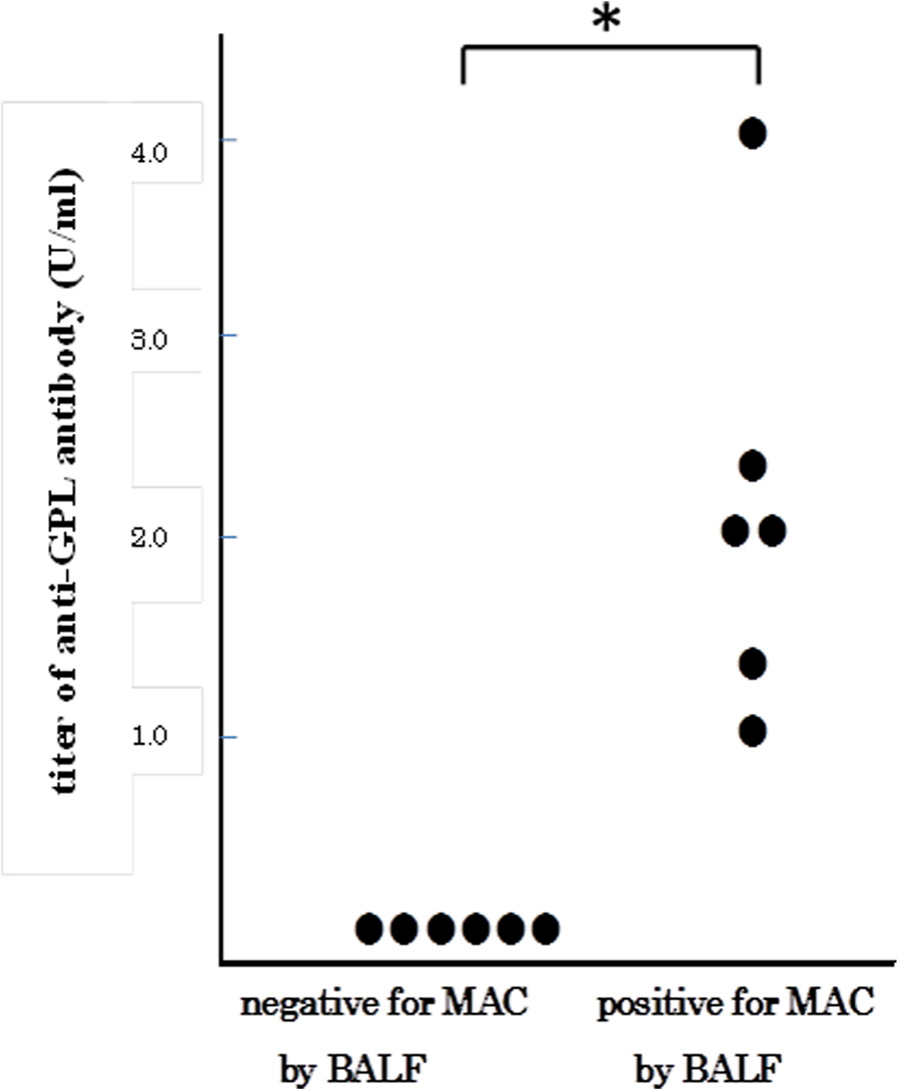
Anti-GPL antibody titers of 12 patients who underwent bronchoscopy. Twelve patients with negative expectorated sputum cultures for MAC and positive CT findings compatible with MAC underwent bronchoscopy. BALF samples from six patients were positive for MAC, and the remainder were negative. Titers of anti-GPL antibodies in BALF samples were compared between the MAC-negative and MAC-positive groups. *P = 0.0039. *BALF* bronchoalveolar lavage fluid, *CT* computed tomography, *GPL* glycopeptidolipid, *MAC Mycobacterium avium* complex

**Fig. 3:**
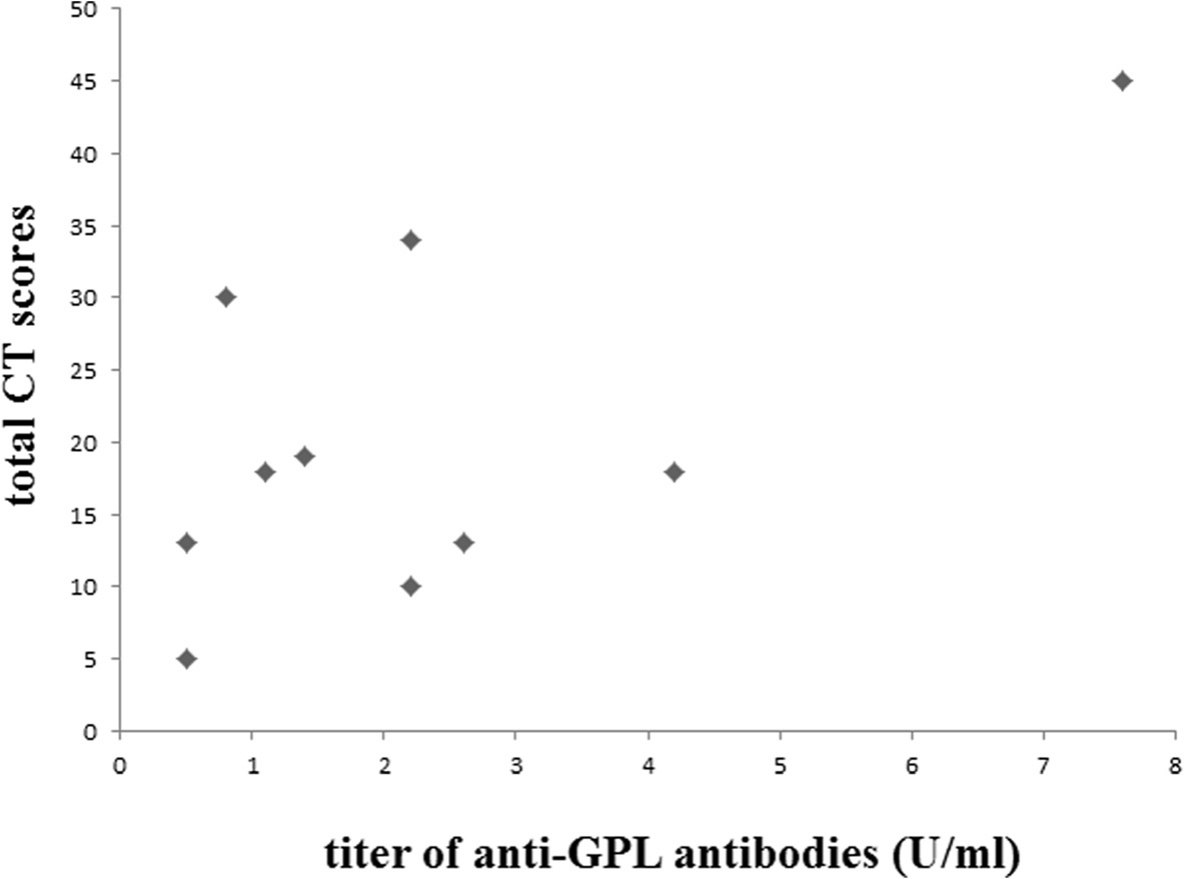
Relationship between anti-GPL antibody titers and radiological severity by computed tomography (CT) in 10 patients with rheumatoid arthritis with *Mycobacterium avium* complex pulmonary disease. A positive correlation was observed between the severity of the disease and titers of the antibodies (*r* = 0.67, *P* = 0.049). *GPL* glycopeptidolipid

Anti-GPL antibodies were detected in the sera of all six of the patients with positive results for MAC-PD by BALF sampling, whereas these antibodies were not detected in the sera of the six patients with negative results for MAC-PD by BALF sampling. Titers of anti-GPL antibodies are shown in Fig. 2. Chest CT images detected bronchiectasis, centrilobular nodules, nodules larger than 5 mm, air space disease, and cavities in 100 %, 70 %, 50 %, 90 %, and 20 % of 10 patients with MAC-PD, respectively. Of the 10 patients with RA with MAC-PD, a positive correlation was observed between the total CT scores of the involved segments, which represented the extent of MAC-PD, and the titers of anti-GPL antibodies (*r* = 0.67, *P* = 0.049) (Fig. 3).

### Comparison of patients with RA with versus without MAC-PD

Baseline data for patients with MAC-PD (n = 10) and without MAC-PD (n = 359) are shown in Table 3. Patients with RA with MAC-PD had a longer disease duration (*P* = 0.028), a lower body mass index (BMI) (*P* = 0.020), and a smaller number of peripheral blood lymphocytes (*P* = 0.044) than patients with RA without MAC-PD. The patients with MAC-PD tended to be older than those without MAC-PD, but no significant difference was noted. The average age of the 369 patients who were measured for anti-GPL antibodies in this study was found to be older than that of the 381 patients who were not screened for this study (64.3 ± 13.0 years versus 61.5 ± 13.1 years).

**Table 3:** Characteristics of patients with rheumatoid arthritis with and without *Mycobacterium avium* complex pulmonary disease

Variable	Overall (n = 369)	RA with MAC-PD (n = 10)	RA without MAC-PD (n = 359)	*P* value^a^
Age (yr)	64.3 ± 13.0	72.2 ± 6.1	64.1 ± 13.1	0.065
Female sex (%)	80.8	80.0	81.2	0.95
BMI (kg/m^2^)	21.8 ± 1.9	**19.2 ± 2.3**	**21.8 ± 3.5**	**0.020**
RA disease duration (yr)	13.0 ± 3.4	**21.0 ± 4.7**	**11.2 ± 3.4**	**0.028**
Current smoking (%)	11.0	0	11.4	0.26
RF positivity (%)	83.8	90.0	83.5	0.60
Steinbrocker stage III or IV (%)^b^	63.6	80.0	61.2	0.24
Steinbrocker class 3 or 4 (%)^b^	12.5	0	13.1	0.22
DAS28-CRP	2.8 ± 1.3	3.0 ± 0.8	2.8 ± 1.7	0.48
Peripheral blood lymphocytes (count/μl)	1736 ± 26.1	**1322 ± 23.6**	**1740 ± 25.5**	**0.044**
Oral corticosteroid use (%)	30.0	30.0	29.0	0.95
Prednisolone dosage (mg/day)	3.2 ± 1.4	3.7 ± 1.2	3.1 ± 1.5	0.56
MTX use (%)	66.7	60.0	67.4	0.62
MTX dosage (mg/wk)	7.5 ± 1.6	5.7 ± 1.5	7.6 ± 2.6	0.075
Use of immunosuppressive drugs, except for MTX (%)^c^	8.2	10.0	7.2	0.74
Use of biologic DMARDs (%)	52.9	60.0	52.4	0.65
Duration of biologic DMARD use (yr)	NA	4.2 ± 2.6	4.0 ± 2.8	0.87
Diabetes mellitus (%)	9.8	20.0	8.9	0.23

*Abbreviations: BMI* body mass index, *DAS28-CRP* Disease Activity Score in 28 joints using C-reactive protein level, *DMARD* disease-modifying antirheumatic drug, *MAC-PD Mycobacterium avium* complex pulmonary disease, *MTX* methotrexate, *NA* not applicable, *RA* rheumatoid arthritis, *RF* rheumatoid factor

Values are the percentage or mean ± standard deviation unless otherwise indicated. Bold values indicate significant differences (*P* < 0.05)

^a^Between patients with RA with and without MAC-PD

^b^Steinbrocker et al. classification system [20] was used to define RA disease stages and classes

^c^Immunosuppressive drugs included tacrolimus, leflunomide, and mizoribine

## Discussion

In the present study, we assessed, for the first time to our knowledge, the clinical usefulness of an EIA kit as a supplementary diagnostic aid for MAC-PD in a large series of patients with RA. Our results revealed that a positive likelihood ratio, pretest probability, and positive and negative posttest probabilities were 80.0, 2.7 %, 69 %, and 0.54 %, respectively. The positive likelihood ratio of 80.0 indicated that detection of serum anti-GPL antibodies has a large effect on increasing the probability of MAC-PD presence. Pre- and posttest probability values showed that the probability of MAC-PD presence increases from 2.7 % to 69 % after obtaining a positive test of serum anti-GPL antibodies. In addition, measuring anti-GPL antibodies showed excellent positive and negative predictive values and AUC for MAC-PD in patients with RA.

Although the specificity of the EIA kit was high, false-positive EIA determinations were obtained in four patients. In these four patients, titers of anti-GPL antibodies were positive at low values (0.7, 0.9, 1.8, and 2.0 U/ml). Several explanations have been proposed for these false-positive results. First, they could have been due to extrapulmonary infections by MAC [15]; however, this was unlikely in our patients because of the lack of clinical symptoms, negative culture results, and negative positron emission tomography findings. Second, they could have been due to asymptomatic colonization with MAC organisms from the environment [8]. Third, they could have been due to non-specific binding of IgA in serum to the GPL core antigen.

The average titer of anti-GPL antibodies in the antibody-positive individuals in a previous study conducted with patients with non-rheumatic disease [6] was 10.7 ± 7.9 U/ml, whereas it was 2.53 ± 1.94 U/ml in our study. The lower titer of anti-GPL antibodies and two false-negative cases observed in patients with RA could be explained by the following reasons: (1) impaired antibody responses against the MAC GPL antigen by immunosuppressive therapy, (2) altered immune responses to GPL core potentially governed by human leukocyte antigen genes [16], and/or (3) less severe MAC-PD in the two false-negative cases. Concomitant use of methotrexate (MTX) may affect the positivity and titers of anti-GPL antibodies. In the present study, the average dosage of MTX used for the enrolled patients was lower than that commonly used in Western countries. Further studies are needed to investigate anti-GPL antibody generation in patients with RA receiving higher dosages of MTX.

The incidence of NTM in the Japanese general population is estimated at 5.7/100,000/yr [17], but no data are available for age-matched prevalence of MAC-PD. Considering the incidence of MAC-PD in Japanese general population, the prevalence of MAC-PD in our study appears to be very high. We speculated that both intensive examinations for MAC-PD among the enrolled patients, comprising CT, sputum culture, and bronchoscopy, and the older age of the patients in the present study might be responsible for this high prevalence.

Differentiating MAC-PD from other pulmonary comorbidities, including RA-associated interstitial lung disease, is challenging in clinical practice. The initial imaging study obtained for diagnosis of pulmonary comorbidity in patients with RA is almost always a chest x-ray. When we analyzed the diagnostic performance of anti-GPL antibodies in 57 patients with RA who had abnormal shadows on their chest x-rays, the positive and negative predictive values and accuracy of the assay were estimated as 100 %, 96 %, and 97 %, respectively. Although the number of the patients was small, these data indicated promising diagnostic performance of anti-GPL antibodies in this patient population. Furthermore, our study demonstrates the usefulness of testing for this antibody in patients with chest CT findings compatible with MAC-PD. MAC organisms were detected in the BALF of all six patients who had positive CT findings compatible with MAC-PD and anti-GPL antibodies, whereas the organisms were not detected in the BALF of the other six patients who had negative anti-GPL antibodies in spite of characteristic CT findings. These results support the possibility that combining positive anti-GPL antibodies and positive CT findings for MAC-PD could yield an alternative diagnostic procedure for MAC-PD, even in patients with negative expectorated sputum culture results for MAC. Further investigations based on a prospective cohort study recruiting patients with RA with abnormal x-ray and CT findings are required to fully establish the usefulness of this antibody detection in differentiating MAC-PD from other pulmonary comorbidities. A cost-effectiveness analysis for measuring anti-GPL antibodies in patients with RA is also needed.

Measuring anti-GPL antibodies could be a useful tool for evaluating disease severity and monitoring disease activity of MAC-PD in addition to its diagnostic ability, based on the following findings. First, the titers of the antibodies showed significant correlation with the extent of MAC-PD in patients with RA, as shown in Fig. 3. Second, we found one patient whose serum level of anti-GPL antibodies gradually declined after successful antimicrobial chemotherapy (Additional file 1), suggesting that the level of the antibodies reflected disease activity of MAC infection. Further investigation is required to establish this relationship in patients with RA.

The results of univariate analysis revealed that patients with RA with MAC-PD had a longer disease duration, a lower BMI, and a smaller number of peripheral lymphocytes (Table 3) than other subjects. However, cross-sectional studies cannot establish causality; they can only determine associations. Therefore, a longitudinal study is required to identify risk factors for development of MAC-PD in patients with RA.

Before initiating immunosuppressive treatment of RA, screening for latent or chronic infectious diseases is mandatory to ensure patient safety and continuation of treatment without infectious events. Current guidelines recommend screening tests for tuberculosis and hepatitis virus in particular [18, 19]. Given the high diagnostic performance of anti-GPL antibodies for diagnosing MAC-PD in patients with RA and the clinical consequences of this pulmonary disease, we recommend measuring anti-GPL antibodies before starting treatment of RA, especially in patients with abnormal chest x-rays in regions or countries with moderate to high prevalence of MAC-PD. Those patients who have positive tests for anti-GPL antibodies should be examined with additional pulmonary imaging and microbiological studies to diagnose MAC-PD in consultation with a pulmonologist.

## Conclusions

Measuring anti-GPL antibodies showed high specificity with preserved sensitivity and excellent positive likelihood ratio, positive and negative predictive values, and AUC for MAC-PD in patients with RA. Screening for MAC-PD using anti-GPL antibodies helps to identify patients who should undergo further examination for diagnosis of MAC-PD. The present study also demonstrates that the titers of anti-GPL antibodies correlated with the extent of MAC-PD in patients with RA. Anti-GPL antibodies in combination with chest radiography and CT may provide a new strategy for diagnosis and evaluation of MAC-PD in patients with RA.

## Additional file

Additional file 1:
**Changes of serum level of anti-GPL antibodies in a MAC-PD patient with RA.** The levels of the antibodies gradually declined after successful antimicrobial chemotherapy with rifampicin, ethambutol (EB), clarithromycin (CAM), and streptomycin (SM). Because of skin rash, the therapeutic regimen was changed to erythromycin (EM). Sputum specimens were successfully converted to negative. (TIFF 30 kb)

## Abbreviations

ab: antibodies
AUC: area under the curve
BAL: bronchoalveolar lavage
BALF: bronchoalveolar lavage fluid
BMI: body mass index
CAM: clarithromycin
DAS28-CRP: Disease Activity Score in 28 joints using C-reactive protein level
DMARD: disease-modifying antirheumatic drug
EB: ethambutol
EIA: enzyme immunoassay
EM: erythromycin
GPL: glycopeptidolipid
HRCT: high-resolution computed tomography
IgA: immunoglobulin A
MAC: *Mycobacterium avium* complex
MTX: methotrexate
NA: not applicable
NTM: nontuberculous mycobacteria
PD: pulmonary disease
RA: rheumatoid arthritis
RF: rheumatoid factor
SM: streptomycin
