# Supplementary figures and images for: Diagnostic performance of measuring antibodies to the glycopeptidolipid core antigen specific to Mycobacterium avium complex in patients with rheumatoid arthritis: results from a cross-sectional observational study
Source: Arthritis Res Ther. 2015 Sep 28;17:273. doi: 10.1186/s13075-015-0787-y (PMC4585998; doi:10.1186/s13075-015-0787-y)

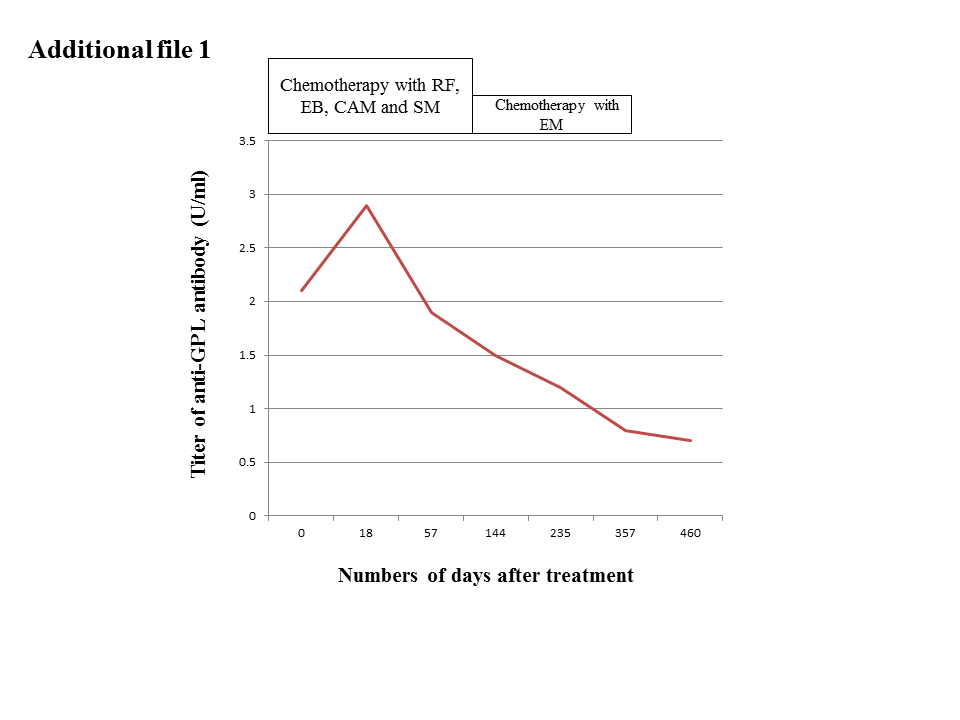

Supplement: Additional file 1: — Changes of serum level of anti-GPL antibodies in a MAC-PD patient with RA. The levels of the antibodies gradually declined after successful antimicrobial chemotherapy with rifampicin, ethambutol (EB), clarithromycin (CAM), and streptomycin (SM). Because of skin rash, the therapeutic regimen was changed to erythromycin (EM). Sputum specimens were successfully converted to negative. (TIFF 30 kb) [file 13075_2015_787_MOESM1_ESM.tiff]
